# Facial Scanning Accuracy with Stereophotogrammetry and Smartphone Technology in Children: A Systematic Review

**DOI:** 10.3390/children9091390

**Published:** 2022-09-14

**Authors:** Vincenzo Quinzi, Alessandro Polizzi, Vincenzo Ronsivalle, Simona Santonocito, Cristina Conforte, Rebecca Jewel Manenti, Gaetano Isola, Antonino Lo Giudice

**Affiliations:** 1 Department of Life, Health & Environmental Sciences, Postgraduate School of Orthodontics, University of L’Aquila, 67100 L’Aquila, Italy; 2 Department of General Surgery and Surgical-Medical Specialties, School of Dentistry, University of Catania, 95124 Catania, Italy

**Keywords:** accuracy, 3D facial scanning, smartphones, stereophotogrammetry

## Abstract

The aim of the study was to systematically review and compare the accuracy of smartphone scanners versus stereophotogrammetry technology for facial digitization in children. A systematic literature search strategy of articles published from 1 January 2010 to 30 August 2022 was adopted through a combination of Mesh terms and free text words pooled through boolean operators on the following databases: PubMed, Scopus, Web of Science, Cochrane Library, LILACS, and OpenGrey. Twenty-three articles met the inclusion criteria. Stationary stereophotogrammetry devices showed a mean accuracy that ranged from 0.087 to 0.860 mm, portable stereophotogrammetry scanners from 0.150 to 0.849 mm, and smartphones from 0.460 to 1.400 mm. Regarding the risk of bias assessment, fourteen papers showed an overall low risk, three articles had unclear risk and four articles had high risk. Although smartphones showed less performance on deep and irregular surfaces, all the analyzed devices were sufficiently accurate for clinical application. Internal depth-sensing cameras or external infrared structured-light depth-sensing cameras plugged into smartphones/tablets increased the accuracy. These devices are portable and inexpensive but require greater operator experience and patient compliance for the incremented time of acquisition. Stationary stereophotogrammetry is the gold standard for greater accuracy and shorter acquisition time, avoiding motion artifacts.

## 1. Introduction

Facial acquisition and 3D imaging are useful in many fields of medicine such as maxillo-facial surgery, the production of prostheses, forensic medicine, and orthodontics [1,2,3]. Precise acquisition of 3D facial scanning incorporated with dental design software may improve treatment planning predictability [1,4,5,6]. Facial anthropometry, which made use of calipers and protractors to measure indices, was traditionally employed for facial acquisition [7]. However facial anthropometry, despite its simplicity, is operator-dependent and time-consuming, potentially inducing discomfort to the patients [8].

2D digital photography may be useful during dental treatment planning, for example, to visualize some measurements used to communicate with the patients. However, it is not possible to simulate a 3D object such as the human face; therefore, 2D photography is not suitable for the detection of facial volumes, deformities, and asymmetries [4,8,9].

Modern technologies overcame the limitations of direct anthropometry and 2D photography. Recently, 3D optical facial scanners have been commercialized with the aim of the acquisition of 3D patient facial images [10]. A facial scanner may provide a reliable method of facial digitization in order to create a virtual patient for treatment planning. The different 3D facial scanning technologies can be classified as follows: photogrammetry, stereophotogrammetry [11,12,13,14,15,16], laser beam scanning [9,17,18], structured light scanning [19,20], and dual structured light with infra-red sensor. Photogrammetry and stereophotogrammetry are passive scanning systems consisting of taking two or more photos from different perspectives; the 3D point cloud is then obtained from the common points through a reverse engineering software program. On the contrary, laser beam scanning and structured light scanning are active scanning systems; more specifically, light patterns projected at the patient are detected by a camera to obtain the 3D point cloud [19,20,21,22,23].

Stereophotogrammetry has been one of the most employed face scanning acquisition systems [24], which is fast (rapid capture of the face’s shape and texture), non-invasive, and produces accurate 3D images [4,25,26]. However, most professional stereophotogrammetry scanners are complex and expensive, and often require a long learning curve to optimally perform the scanning protocols [27,28,29,30]. Recently, interest in the use of smartphones with 3D depth sensor cameras that use structured light or time of flight technology for face scanning is spreading. These devices have the advantage of being portable, inexpensive, and popular [31,32,33,34]. Other smartphone scanning advantages include reduced time consumption for scanning image processing, and technical learning [35]. Different smartphone applications have been created for face digitalization in order to obtain 3D facial models transferable to dental computer-aided design (CAD) software [31,33].

Facial scanning is more challenging in children for different reasons. First of all, they may be uncooperative patients. Facial scanning, to be optimal, requires the subject to remain still for the entire time of acquisition and this may not be easy with a child. Furthermore, in the case of syndromic patients, some current technologies may have greater difficulties in the specific acquisition of any facial deformities. All these factors could affect the accuracy of the facial scan and therefore it is necessary to create new technologies that may overcome these difficulties and make the acquisition time less but with greater diagnostic accuracy. In this regard, close-range photogrammetry integrated with machine learning has been proposed and could be promising to create 3D children’s facial models [36].

The accuracy of these systems is still under evaluation, therefore, the aim of this study is to systematically review and compare, in the light of current knowledge, the accuracy of smartphone scanners versus stereophotogrammetry technology for facial digitization.

## 2. Materials and Methods

### 2.1. Protocol, Registration and Search Strategy

This systematic review followed the Preferred Reporting Items for Systematic Reviews and Meta-analyses (PRISMA) guidelines [37] and it has also been registered on the PROSPERO database (registration number: CRD42021241229). The population, intervention, comparison, and outcomes (PICO) question has been formulated as follows: are extra-oral scannings for the reproduction of a virtual facial model (P) obtained by smartphone technology (I) comparable to those obtained by stereophotogrammetry (C) in terms of accuracy (O)? The search strategy involved a combination of Mesh terms and free text words pooled through boolean operators (‘AND’, ‘OR’) and it has been performed on the following databases: PUBMED, Scopus, Web of Science, Cochrane Library, LILACS, and OpenGrey (Table 1).

### 2.2. Inclusion, Exclusion Criteria and Outcomes

The studies included in this systematic review were: randomized and non-randomized controlled trials, cohort studies, case-control studies, cross-sectional studies, retrospective studies, and thesis, only in English language and for a publication period from 1 January 2010 to 30 August 2022. Case reports, opinion articles, and reviews were excluded. The population of interest involved human faces or objects reproducing the human face; in particular, 3D virtual facial models obtained from optical facial scanners based on stereophotogrammetry or smartphone technology, but the studies with the use of 2D model images only were excluded. The main outcome of this systematic review consisted of the accuracy of facial measurements obtained by stereophotogrammetry versus smartphone applications scannings. Accuracy is intended as the discrepancy between the virtual facial model obtained through the scanner and a reference model. The deviation was measured in terms of inter-landmarks linear distances or surface-to-surface deviations.

### 2.3. Study Selection and Data Extraction

Records identified through database searches underwent an initial screening (title and abstract evaluation) where potential relevant articles were selected. The eligible articles underwent a further review in the full-text versions for adherence to the inclusion criteria. The data extracted from the studies included in the study are: authors, year of publication, aim of the study, characteristics of the sample and of the scanning method, standard reference for validation, characteristics of the measurements carried out, and conclusions. Disagreements between the review authors over the selection of particular articles were resolved by discussion, with involvement of a third review author, where necessary.

### 2.4. Risk of Bias Assessment

Two reviewers independently assessed the risk of bias in included studies using the Quality Assessment Tool for Diagnostic Accuracy Studies-2 (QUADAS-2) [38], which comprises 4 domains: patient selection, index test, reference standard, and flow and timing. When 1 or more of the key domains are scored as high risk, the study in question is considered with a high risk of bias. When more than 2 key domains are judged as unclear, the study is regarded with an unclear risk of bias. Disagreements between the review authors over the risk of bias in particular articles were resolved by discussion, with involvement of a third review author where necessary.

## 3. Results

### 3.1. Study Selection

**Figure 1:**
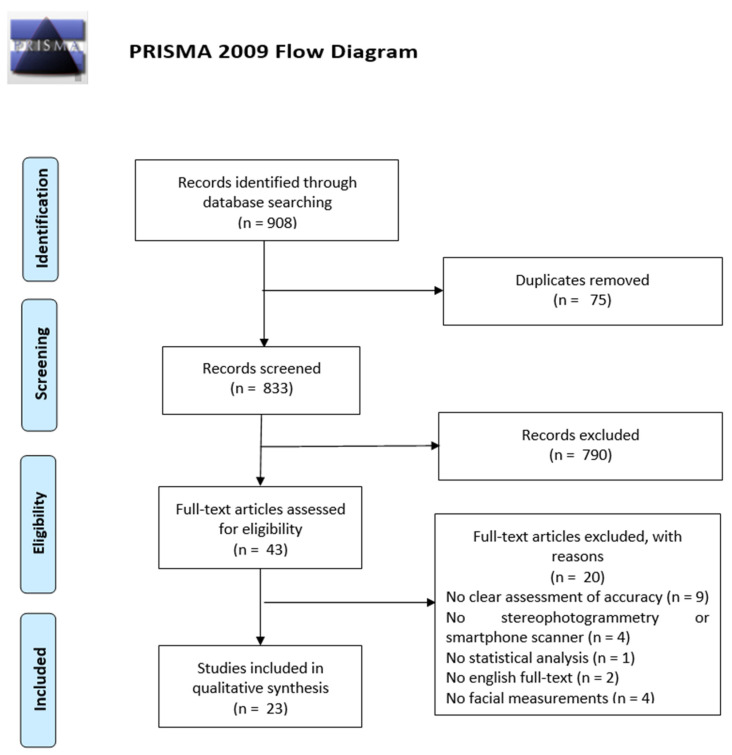
PRISMA flow diagram of the searching strategy and results.

The database searching led to the identification of 908 articles: 244 from Pubmed, 215 from Scopus, 320 from Web of Science, 102 from Cochrane Library, 25 from LILACS, and 2 from OpenGrey (Table 1). 75 duplicates were removed through the title review and, after the abstract screening of the 833 remaining articles, 43 full-text studies were assessed for eligibility. 23 articles were considered as eligible for the review analysis, whereas the other 20 papers were excluded for: no clear assessment of accuracy, no stereophotogrammetry or smartphone scanner, no statistical analysis, no English full-text, and no facial measurements. The PRISMA flow diagram of the search and evaluation process is illustrated in Figure 1.

### 3.2. Risk of Bias and Applicability Concern

**Figure 2:**
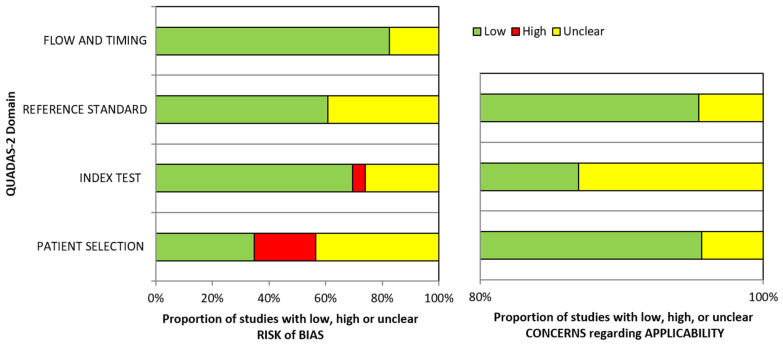
Weighted barchart for the risk of bias and application concerns of the selected studies according to the QUADAS-2 guidelines.

Table 2 shows the risk of bias and application concerns according to the QUADAS-2 guidelines. Among 23 articles [39,40,41,42,43,44,45,46,47,48,49,50,51,52,53,54,55,56,57,58,59,60,61], 14 papers showed an overall low risk of bias [40,41,43,45,46,47,48,49,50,51,53,56,59,60,61], 3 articles had unclear risk of bias [39,44,52,57] and 4 articles displayed a high risk of bias [42,54,55,58]. Concerning applicability concerns, all articles showed an overall low level of concern, however, the domain “index test” showed more frequently an unclear concern because some papers did not sufficiently describe the scanning procedures and the used devices [40,46,55]. Regarding the quality assessment, the domain “patient selection” showed the highest risk of bias because of the exclusion of maxillofacial abnormalities [42,45,54,55,58] (this may result in overestimation of diagnostic accuracy [38]), the small number of participants [40,41,46,51,52] and the unclear method of random sampling [40,51,57]. Weighted bar chart for the risk of bias and application concerns of the selected studies is shown in Figure 2.

### 3.3. Description of the Included Studies

The included studies’ characteristics are shown in Table 3. Among the 23 studies, 17 involved adult volunteers [39,42,43,44,45,48,49,50,53,54,55,56,57,58,59,60,61] (the range of the number of participants was 5–80). The other 6 studies were conducted on impression casts of the face [41,46,51], mannequin heads [40,52], and human cadaver heads [47]. However, 2 studies [48,49] included both volunteers and mannequin heads.

In terms of tested scanning methods, all included studies analyzed stereophotogrammetry, smartphone scanning, or both. More specifically, thirteen articles tested the accuracy of stereophotogrammetry [41,42,43,45,47,49,50,51,52,56,59,60,61], six articles a smartphone structured light scanner [39,40,43,44,57,58], three articles smartphone photogrammetry [46,54,56],two articles a portable dual-structured light scanner connected to a tablet [52,55], seven articles one or more laser scanners [40,41,42,46,47,48,58], three articles a structured light scanner [53,58,59], three articles computed tomography [40,47,61] and two articles 2D camera photogrammetry [42,45].

Furthermore, the most used reference method (which generated the reference model) was direct anthropometry [40,42,43,45,47,50,55,58,59], followed by stereophotogrammetry [39,44,48,49,51,52,53,57], structured light scanner [54,56] and computed tomography [41,46]. In one paper, a reference model generated by a laser scanner was used [60] and another study used a coordinate-measuring machine as the gold standard [61].

The facial landmarks considered in the articles ranged from 6 to 29. The linear distances and angles analyzed ranged, respectively, from 5 to 25 and from 3 to 17. In one study [53], two lego bricks attached to participants’ faces were employed as a reference object to measure the scanner accuracy. Most of the included articles measured the global surface-to-surface deviation between the reference and test images obtained from the scannings [40,41,46,48,49,53,54,56,57,60].

## 4. Discussion

The aim of this review was to investigate the accuracy of smartphone scanners to generate digital face models compared to stereophotogrammetry. Compared to a previous systematic review [62] on face scanning, our study specifically analyzed the effectiveness of modern systems integrated into smartphones, comparing them with stereophotogrammetry. The selection of the articles was carried out on a greater number of databases (including gray literature). Furthermore, this systematic review included a greater number of selected articles (11 vs. 23) and the literature search is updated to August 2022 (vs. May 2020). Finally, the previous review included four articles concerning the use of smartphones/tablets, while ours included eleven studies.

Stereophotogrammetry is a passive scanning system that consists of capturing face surfaces through multiple photoshoots all taken at the same time from different perspectives. The 3D digital face model consisting of a dense cloud of points is obtained through software that uses the information of the camera’s position (with set angles and distances) and camera-to-subject distance [29,63]. This technology is able to reproduce very realistic and colored face models, but the accuracy is highly dependent on the camera’s resolution and the light conditions [41,64]. This system requires the application of standardized flash units to eliminate interference from ambient light and the careful assessment of camera settings such as brightness level, aperture, and shutter speed [29]. All this requires expensive investments and specific skills to be acquired.

Thirteen included articles reported the results of stationary stereophotogrammetry scanners to reproduce 3D facial models [39,41,44,45,47,48,49,50,52,53,59,60,61]. Among these, ten studies concerned the accuracy of stationary stereophotogrammetry systems [39,44,45,47,48,52,53,59,60,61]. All the articles concluded that this system is really reliable for reproducing digital face models with an accuracy comparable or superior to other systems such as direct anthropometry, CMM, laser, and structured light scanners. However, not all stationary stereophotogrammetry systems showed the same accuracy and the reference method used for comparison differed through the included articles.

The other three included articles that compared the accuracy of stationary and portable stereophotogrammetry scanners [41,49,50]. Two studies reported that portable stereophotogrammetry scanners (Scanify and Vectra H1) showed high accuracy, but lower compared to stationary devices (Danae 100SP, 3dMDface, and Vectra M3); moreover, portable scanners suffered more the influence of involuntary facial movements [41,49]. However, in a similar study conducted by Kim et al. [50], portable Vectra M1 and stationary Vectra M3 yielded similar values. The contrasting results (despite the use of the same devices, in particular for the study of Gibelli et al. [49]), may be explained by the different number of participants, anthropometric landmarks, methodology, and statistical analysis adopted. Therefore, portable stereophotogrammetry scanners showed to be reliable for clinical use, however, further investigations must be conducted to better understand the limitations and advantages of handled stereophotogrammetry scanners.

Two other articles evaluated selectively, the accuracy of portable stereophotogrammetry scanners [42,51]. Dindaroglu et al. [45] reported that Vectra H1 showed higher accuracy and reliability compared to 2D photography and a laser scanner for the morphological evaluation of soft tissues. Liu et al. compared a very low-cost stereophotogrammetry portable scanner (Scanify) to Vectra H1 and the authors concluded that the first device needs future improvements for clinical application [51].

In the included studies we found that stationary stereophotogrammetry devices showed a mean accuracy that ranged from 0.087 to 0.860 mm, portable stereophotogrammetry scanners from 0.150 to 0.849 mm, and smartphones/tablets from 0.460 to 1.400 mm (2D photogrammetry reported the highest values) (Table 4). A digital face scanner can be considered:

- highly reliable, if mean accuracy is <1.0 mm,
- reliable, if mean accuracy is 1.0–1.5 mm,
- moderately reliable, if mean accuracy is 1.5–2.0 mm,
- unreliable, if mean accuracy is >2.0 mm [65].

**Table 4:** Results of the scanners’ mean accuracy (expressed in millimeters) in the selected studies. NA: not available. * SD not reported; ^1^ Stationary stereophotogrammetry; ^2^ Portable stereophotogrammetry; ^a^ smartphone 3D scanner; ^b^ 2D photogrammetry; ^c^ tablet connected to a portable dual-structured light scanner; P: photographs.

Study	Stereophotogrammetry (Mean ± SD)	Smartphone (Mean ± SD)	Structured Light Scanner (Mean ± SD)	Laser Scanner (Mean ± SD)	2D Camera (Mean ± SD)
Akan et al. (2022) [39]	-	iPhoneX ^a^: 0.753 ± 0.113	-	-	-
Amornvit et al. (2019) [40]	-	iPhoneX ^a^: NA	-	EinScan Pro: NA EinScan Pro 2X Plus: NA	-
Aswehlee et al. (2018) [41]	Danae^1^: 0.087 ± 0.002 3dMDface ^1^: 0.123 ± 0.007 Scanify ^2^: 0.849 ± 0.046	-	-	Vivid 910: 0.068 ± 0.001	-
Ayaz et al. (2020) [42]	Vectra H1 ^2^: 0.280 *	-	-	Planmeca ProFace: 0.500 *	Nikon D800 2D camera: 0.780 *
Chong et al. (2021) [43]	Vectra H1 ^2^: NA	iPad/iPhone ^b^: NA	-	-	-
D’Ettorre et al. (2022) [44]	-	iPhoneXs ^b^ (Bellus3D Face App): NA iPhoneXs ^b^ (Capture App): NA	-	-	-
Dindaroglu et al. (2016) [45]	3dMDface ^1^: NA	-	-	-	Canon EOS 40D 2D camera: NA
Elbashti et al. (2019) [46]	-	iPhone6 ^b^: −24P (0.605 ± 0.124)	-	Vivid 910: 0.068 ± 0.001	-
Fourie et al. (2011) [47]	Di3D ^1^: 0.860 ± 0.570	-	-	Vivid 900: 0.890 ± 0.560	-
Gibelli et al. (2018) [48]	Vectra M3 ^1^: 0.650 ± 0.120	-	-	Sense: 0.420 ± 0.170	-
Gibelli et al. (2018) [49]	Vectra H1 ^2^: 0.440 ± 0.360 Vectra M3 ^1^: 0.220 ± 0.140	-	-	-	-
Kim et al. (2018) [50]	Vectra H1 ^2^: NA Vectra M3 ^1^: NA	-	-	-	-
Liu et al. (2019) [51]	Vectra H1 ^2^: 0.15 ± 0.015 Scanify ^2^: 0.740 ± 0.089	-	-	-	-
Liu et al. (2021) [66]	3dMDface ^1^: 0.36 ± 0.20	Face Camera Pro Bellus ^c^: 0.61 ± 0.47	-	-	-
Modabber et al. (2016) [53]	FaceScan 3D ^1^: 0.523 ± 0.144	-	Artec EVA: 0.228 ± 0.051	-	-
Nightingale et al. (2020) [54]	-	iPhone8S ^b^: −40P (0.800 ± 0.200) −60P (0.900 ± 0.400) −80P (0.800 ± 0.300)	Artec Spider: NA	-	-
Piedra-Cascon et al. (2020) [55]	-	Face Camera Pro Bellus ^c^: 0.910 ± 0.320	-	-	-
Ross et al. (2018) [56]	-	iPhone7 ^b^: −30P (1.200 ± 0.300) −60P (1.200 ± 0.200) −90P (1.400 ± 0.600)	Artec Spider: NA Intel RealSense Camera SR300: 1.800 ± 0.300	-	-
Rudy et al. (2020) [57]	Vectra H1 ^2^: NA	iPhoneX ^a^: 0.460 ± 0.010	-	-	-
Wang et al. (2022) [58]	-	iPad Pro 2020 ^b^: 1.17 ± 0.80	ARC-7 Face Scanning System: 0.76 ± 0.61	EinScan Pro 2X Plus: 0.69 ± 0.65	-
Ye et al. (2016) [59]	3dMDface ^1^: 0.620 ± 0.390	-	3D CaMega: 0.580 ± 0.370	-	-
Zhao et al. (2017) [60]	3dMDface ^1^: 0.580 ± 0.110 FaceScan3D ^1^: 0.570 ± 0.070	-	-	Faro LLP: NA	-
Zhao et al. (2021) [61]	3dMD face ^1^: NA	-	-	-	-

In clinical practice, facial models with deviations < 1.5 mm can be considered acceptable [59,60,67]. Therefore, accurate digital face models are reproduced from both stereophotogrammetry and smartphone scanning technology; however, smartphones seem to be less accurate as reported in two studies, in particular in measuring depth [40,46]. Therefore, a more inaccurate reproduction of the anatomically complex surfaces of the face than the flat ones must be expected, and it is not known whether this could imply an alteration in terms of clinical applications.

At first, the smartphone scanning method was based on a 2D photogrammetry approach in which the 3D model was produced from the matching of several photographs from different perspectives through a smartphone app [43,46,54,56]. However, differently from smartphone cameras, stereophotogrammetry usually makes use of digital single-lens reflex cameras with higher ISO settings, better noise reduction software, and higher pixel densities [68]. Five included studies evaluated smartphone/tablet 2D photogrammetry using different iPhone/iPad models [43,46,54,56,58]. Elbashti et al. (2019) [46] reported a good mean accuracy (0.605 ± 0.124 mm) for digitizing an impression cast of a facial defect, using a 24P (photographs) photogrammetry protocol. However, a commercially available laser scanner showed higher accuracy (0.068 ± 0.001 mm) in reference to standard computed tomography imaging. In the study of Nightingale et al. (2020) [54] 20 participants’ faces were scanned by novice operators. The reported accuracies for 40P, 60P, and 80P photogrammetry protocols were 0.800 ± 0.200 mm, 0.900 ± 0.400 mm, and 0.800 ± 0.300, all values that indicate a reliable accuracy. Ross et al. [56] reported a mean discrepancy of 1.200 ± 0.300 mm, 1.200 ± 0.200 mm, and 1.400 ± 0.600 mm with 30P, 60P, and 90P photogrammetry protocols. These higher values, but still <1.5 mm, could be due to the fact that an anatomically complex structure in the participants (ear) was selectively scanned. In any case, these results demonstrated that the ear can be scanned quite accurately using iPhone photographs. Recently, Chong et al. (2021) [43] developed an application enabling patients to capture their 3D facial images. Compared to a portable stereophotogrammetry Vectra H1 device, the measurements obtained with the iPhone/iPad application were also significantly correlated to the direct anthropometric measurements. However, the authors observed that the subnasale area needs improvement with that method. Similarly, Wang et al. (2022) [58] detected a sufficient reliability with the use of an app in the iPad Pro 2020 device, however, this system was less accurate compared to a portable laser scanner and a stationary structured light device.

Recently, to improve scanning accuracy (specifically for anatomically more complex surfaces), infrared structured-light depth-sensing cameras have been incorporated into smartphone devices [69]. The working principle of 3D depth-sensing cameras is similar to professional laser scanners and consists of the time-of-flight technique: the sensor array detects the time interval for infrared light to travel to the object and return to the sensor [70,71,72]. However, professional laser scanning systems have high-tech sensors more sensitive to depths [46,73]. Four included articles in this review evaluated digital face model accuracy through iPhone X/iPhone Xs incorporated scanner [39,40,44,57]. Amornvit et al. (2019) [40] analyzed the face scans obtained from a mannequin head and reported that the iPhone X obtained the fastest scan (0.57 min, in contrast to 6.7 and 9.4 min of the professional laser scanners) but it showed less accuracy, in particular in recording depths > 2 mm for which this technology should be not recommended according to the authors. This may be due to the failure of passing the light into the depth during scanning. In contrast, in a study of 16 participants, Rudy et al. (2020) [57] compared the iPhone X scanner to a portable stereophotogrammetry scanner (Vectra H1) used as a reference method. They reported a reliable mean accuracy (0.460 ± 0.010 mm) with the iPhone X scanner. The different results of these two studies may be explained by the different reference methods (respectively, direct anthropometry and stereophotogrammetry), the different methodology, scanner involved, and scanned objects (respectively, one mannequin head and sixteen participants). More recently, Akan et al. (2021) [39], as reported by the study of Amornivit et al., found that the iPhone X device with a depth-sensing camera is quite accurate compared to stationary stereophotogrammetry, but complex structures visualization needs improvements. The most recent included publication on the accuracy of these devices [44] reported a good reliability of the iPhone Xs with two different applications compared to the 3dMD system. However, portable smartphones, although less expensive, need operator accuracy and patient compliance for the incremented time of acquisition.

Finally, external infrared structured-light depth-sensing cameras can be plugged into smartphones, tablets, or laptop computers [74,75,76,77]. Two included studies reported the use of this approach [52,55]. Piedra-Cascon et al. (2020) [55] evaluated the accuracy of a dual structured-light scanner connected to a tablet in reproducing 3D facial models of 10 participants, using direct anthropometry as a reference method. They reported a mean accuracy of 0.910 ± 0.320 mm which is considered acceptable for virtual treatment planning. Similarly, in the recent article of Liu et al. (2021) [52], the Face Camera Pro Bellus system connected to a tablet and stationary stereophotogrammetry both showed a good accuracy and precision for clinical purposes compared to direct anthropometry. However, the use of external structured-light scanners implies that the overall accuracy should be interpreted as a result that includes the performance of the compatible mobile device, therefore, the accuracy should be evaluated for each combination of external scanner and mobile device [62].

One of the main limitations of portable face-scanning systems is motion artifacts that are induced by involuntary facial movements and showed to be the main source of error in the results of these scanners [49,77,78,79]. Therefore, diagnostic accuracy studies on these devices must be conducted on human living subjects for a correct assessment. The use of devices that capture the information with a single scan tends to be naturally more effective for this reason and is especially suitable for children and patients with special needs (Table 5).

Currently, imaging methods and new technologies are moving forward very quickly. New methods are emerging proposing the integration of target-based close-range photogrammetry and facial landmark machine learning detection through smartphone-based approaches [36]. Other promising systems are based on the Simultaneous Localization and Mapping (SLAM) technique and moving camera. This new technology allows the detection of the object’s real-time position through the creation of 3D point clouds. The advantage of this approach is the ability to obtain a high-resolution real-time 3D point with a reduced scanning capture time [80].

This review included only the most recent literature on the topic, regarding publications subsequent to 2010, because of rapid technological changes, but is limited to English publications. We observed a great heterogeneity in the adopted methodology for diagnostic accuracy studies. For example, in most included articles direct anthropometry was the reference method, thereby limiting the measurements practically only to Euclidean distances. Some studies were conducted in vitro and therefore the accuracy of reported values may be overestimated. For research that aims to study the clinical applicability of devices, living persons must be used to include the possibility of motion artifacts, especially for portable devices. Some studies employed novice operators without scanning experience, while others did not specify the operators’ experience or used experienced operators. Furthermore, a diffuse risk of bias in the selected studies was the exclusion of maxillofacial abnormalities that may result in an overestimation of diagnostic accuracy.

## 5. Conclusions

All the analyzed devices showed sufficiently reliable accuracy for clinical application. Stationary stereophotogrammetry scanners, followed by portable ones, showed higher accuracy than smartphones, particularly in the reproduction of complex anatomies.

Different factors affected the accuracy of facial scanning. Stereophotogrammetry showed itself to be highly reliable, however, the quality of the 3D images is dependent on the camera’s resolution, pixel integrity, and the light conditions during photo acquisition. A direct light may induce a glare effect that affects the quality of the acquisition. Therefore, positioned flash units and camera settings must be standardized to perform a good image exposure. On the other hand, smartphones seemed to be less accurate in the acquisition of irregular face surfaces, but the use of depth-sensing cameras may improve 3D image quality. Motion artifacts, induced by involuntary movements, may significantly affect facial scanning accuracy. For this reason, the use of smartphones or other portable scanners may be less suitable for children and uncooperative patients compared to devices with a single scan acquisition. However, new technologies involving the integration of machine learning or SLAM technique and moving camera may be promising to overcome these limitations and perform higher quality 3D face scannings.

The studies included in the review showed that the use of smartphones and tablets is currently practicable in the clinic for 3D facial scanning. The big advantages are the low cost and portability. However, compared to other professional devices, they require greater attention from the clinician and greater compliance by the patient who must be able to remain motionless for the entire duration of the acquisition time of the photos from different perspectives (risk of motion artifacts). The use of devices with internal depth-sensing cameras or external infrared structured-light depth-sensing cameras plugged into smartphones/tablets showed higher accuracy compared to the classic 2D photogrammetry matching approach through the use of some applications. In general, it has been reported that all devices are quite reliable for clinical practice, however, portable and especially stationary stereophotogrammetry remains the gold standard for greater accuracy in detecting deep and irregular surfaces and for shorter acquisition time. Finally, the various limitations and biases of the included articles may have led to an overestimation of the real accuracy of these devices.

## Figures and Tables

**Table 1:** Searching strategy in the different databases.

Database	Boolean Operator	Results
Pubmed	(“virtual patient” OR “virtual face” OR “digital facial models” OR “3D virtual facial model”) AND (“scanner” [MeSH Terms] OR “digital face scan” OR “facial scan” OR “3D face scanning” OR “facial digitalization” OR “stereophotogrammetry” OR “smartphone scanner” OR “smartphone face scanning” OR “smartphone application”) AND (“accuracy” OR “trueness” OR “precision” OR “3D comparison”).	244
Scopus	(“virtual patient” OR “virtual face” OR “digital facial models” OR “3D virtual facial model”) AND (“scanner” OR “digital face scan” OR “facial scan” OR “3D face scanning” OR “facial digitalization” OR “stereophotogrammetry” OR “smartphone scanner” OR “smartphone face scanning” OR “smartphone application”) AND (“accuracy” OR “trueness” OR “precision” OR “3D comparison”).	215
Web of Science	TS = ((virtual face OR digital facial models OR 3D virtual facial model OR digital face OR face) AND (scanner OR digital face scan OR facial scan OR 3D face scanning OR facial digitalization OR face digitalization OR stereophotogrammetry OR smartphone scanner OR smartphone face scanning OR smartphone application) AND (accuracy OR trueness OR precision OR 3D comparison)).	320
Cochrane Library	“virtual patient” OR “virtual face” OR “digital facial models” OR “3D virtual facial model” AND “scanner” OR “digital face scan” OR “facial scan” OR “3D face scanning” OR “facial digitalization” OR “stereophotogrammetry” OR “smartphone scanner” OR “smartphone face scanning” OR “smartphone application” AND “accuracy” OR “trueness” OR “precision” OR “3D comparison”.	102
LILACS	(digital facial models) AND (scanner OR stereophotogrammetry OR smartphone face scanning) AND (accuracy).	25
OpenGrey	(virtual face OR digital facial models OR 3D virtual facial model OR digital face OR face) AND (scanner OR digital face scan OR facial scan OR 3D face scanning OR facial digitalization OR face digitalization OR stereophotogrammetry OR smartphone scanner OR smartphone face scanning OR smartphone application) AND (accuracy OR trueness OR precision OR 3D comparison).	2
Total		908

**Table 2:** Risk of bias and application concerns according to the QUADAS-2 guidelines. 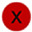 High risk, 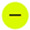 Unclear risk, 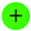 Low risk.

Study	Risk of Bias					Applicability Concerns			
	*Patient selection*	*Index test*	*Reference Standard*	*Flow and Timing*	***Overall risk of bias***	*Patient selection*	*Index test*	*Reference Standard*	***Overall risk of bias***
Akan et al. (2021) [39]	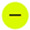	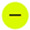	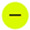	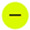	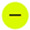	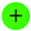	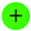	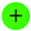	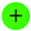
Amornvit et al. (2019) [40]	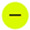	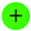	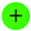	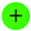	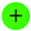	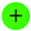	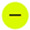	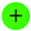	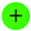
Aswehlee et al.(2018) [41]	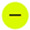	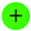	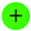	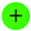	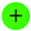	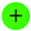	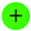	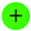	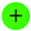
Ayaz et al. (2020) [42]	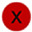	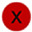	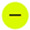	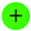	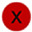	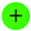	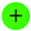	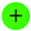	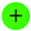
Chong et al. (2021) [43]	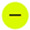	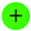	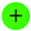	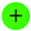	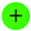	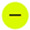	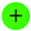	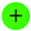	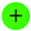
D’Ettorre (2022) et al. [44]	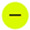	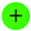	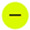	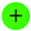	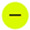	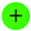	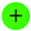	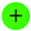	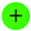
Dindaroglu et al. (2016) [45]	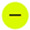	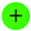	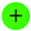	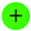	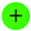	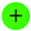	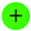	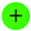	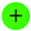
Elbashti et al. (2019) [46]	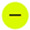	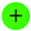	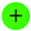	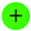	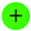	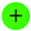	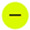	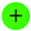	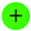
Fourie et al. (2011) [47]	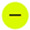	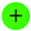	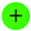	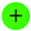	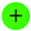	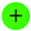	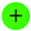	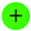	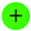
Gibelli et al. (2018) [48]	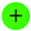	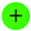	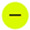	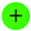	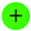	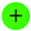	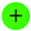	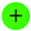	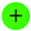
Gibelli et al. (2018) [49]	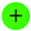	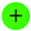	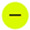	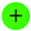	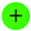	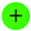	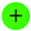	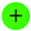	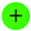
Kim et al. (2018) [50]	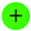	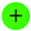	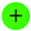	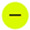	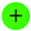	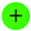	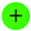	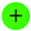	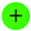
Liu et al. (2019) [51]	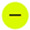	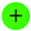	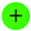	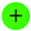	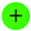	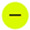	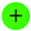	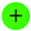	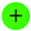
Liu et al. (2021) [52]	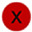	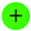	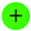	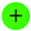	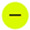	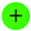	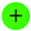	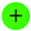	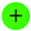
Modabber et al. (2016) [53]	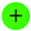	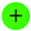	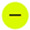	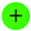	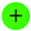	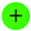	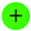	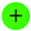	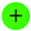
Nightingale et al. (2020) [54]	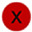	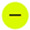	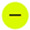	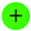	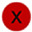	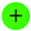	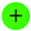	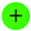	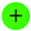
Piedra-Cascon et al. (2020) [55]	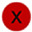	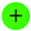	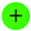	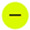	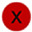	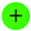	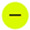	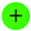	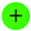
Ross et al. (2018) [56]	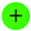	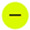	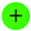	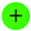	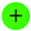	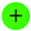	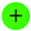	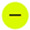	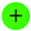
Rudy et al. (2020) [57]	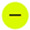	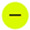	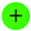	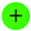	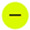	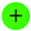	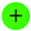	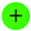	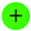
Wang et al. (2022) [58]	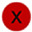	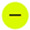	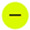	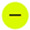	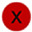	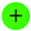	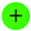	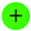	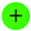
Ye et al. (2016) [59]	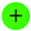	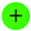	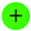	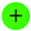	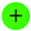	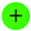	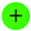	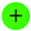	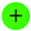
Zhao et al. (2017) [60]	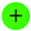	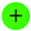	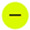	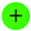	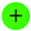	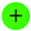	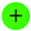	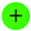	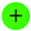
Zhao et al. (2021) [61]	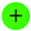	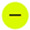	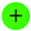	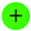	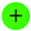	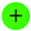	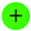	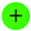	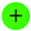

**Table 3:** Characteristics of the included studies. IC: impression cast, IPX: iPhone X; MH: Mannequin head; NA: not applicable; SPG: stereophotogrammetry. Stationary Stereophotogrammetry ^a1^/Portable Stereophotogrammetry ^a2^; Smartphone structured light scanner ^b1^/photogrammetry ^b2^; Tablet connected to a portable dual-structured light scanner ^b3^; Laser scanner ^c^; Structured light scanner ^d^; Computed tomography ^e^; RMSE: root-mean-square error (surface-to-surface).

Study	Aim	Tested Method	Sample	Reference Method	N° Landmarks	Measurements (Metrics)	Conclusions
Akan et al. (2021) [39]	To analyze the accuracy of a depth sensor smartphone camera with conventional stationary stereophotogrammetry	iPhone X with depth-sensing camera ^b1^	26 participants (16 F, 10 M)	3dMD face ^a1^	9	7 linear distances, 3 angles (mean deviations, RMSE)	Depth sensor smartphone camera may be employed for 3D facial scanning. However, complex structures visualization needs improvements.
Amornvit et al. (2019) [40]	To compare the facial scannings acquired through 4 scanner systems with the measurements obtained with direct anthropometry.	EinScan Pro (EP) ^c^, EinScan Pro 2X Plus (EP+) ^c^, iPhone X (IPX) ^b1^, Planmeca ProMax 3D Mid (PM) ^e^	1 MH	Direct anthropometry	NA	Δ*x*, Δ*y*, and Δ*z* (mean linear deviations)	Mean Δ*x* and Δ*y* measurements ranged respectively 10–50 mm and 50–120 mm. The records in the *z*-axis were not possible to acquire. EP+ was the most accurate, EP the intermediate, whereas IPX and PM showed less accuracy. Furthermore, EP, IPX, and PM were less accurate in measuring depths of 2 mm.
Aswehlee et al. (2018) [41]	To evaluate the reliability of different digitalization systems for capturing facial defects.	Vivid 910 ^c^, Danae 100SP ^a1^, 3dMD face ^a1^, Scanify ^a2^.	1 IC	Toshiba TOSCANER-30000μCM ^e^	3D point clouds	Surface deviation (RMSE)	All systems were sufficiently reliable. Laser-beam, light-sectioning technology showed the best accuracy.
Ayaz et al. (2020) [42]	To evaluate the reliability of 2D photography and 3D scanning systems for facial digitalization.	Nikon D800 2D camera, Planmeca ProFace ^c^, Vectra H1 ^a2^	50 participants (25 M, 25 F)	Direct anthropometry	22	7 linear distances, 17 angles (linear and angular mean deviation)	Stereophotogrammetry showed the best accuracy for facial digitalization compared to 2D photography and laser scanner.
Chong et al. (2021) [43]	To evaluate the accuracy of an iPad/iPhone application enabling patients to capture their 3D facial images compared to direct anthropometry.	iPad/iPhone camera through the application “MeinXuan” ^b2^, Vectra H1 ^a2^	20 participants	Direct anthropometry	18	21 linear distances (RMSE, mean absolute difference and relative error measurement)	The measurements obtained with the iPhone/iPad application were significantly correlated to the direct anthropometric measurements. The iPhone/iPad and Vectra H1 mean RMSE were 0.08 and 0.67 mm respectively. The subnasale area needs improvement with the proposed method.
D’Ettorre et al. (2022) [44]	To compare the accuracy of 3D face scanning from 3D stereophotogrammetry and two different applications in smartphones supporting the TrueDepth system.	iPhone Xs equipped with Bellus3D Face or Capture applications ^b1^	40 participants (27 F, 13 M)	3dMD face ^a1^	13	Δ*x*, Δ*y*, and Δ*z* (mean linear deviations)	Stationary stereophotogrammetry is a reliable and fast system. The smartphone applications showed also a good accuracy, are portable and less expensive. However, they need operator accuracy and patient compliance for the incremented time of acquisition.
Dindaroglu et al. (2016) [45]	To compare the stereophotogrammetry accuracy with the direct manual and digital photogrammetry methods.	Canon EOS 40D 2D camera, 3dMD face ^a1^	80 participants (38 M, 42 F)	Direct anthropometry	15	10 linear distances, 6 angles (mean linear and angular deviations)	3D stereophotogrammetry performed accurate 3D facial images reliable in orthodontics.
Elbashti et al. (2019) [46]	To evaluate the accuracy of a smartphone application as a low-cost approach for digitizing a facial defect for 3D modeling.	Vivid 910 ^c^, iPhone 6 ^b2^ (24 photographs)	1 IC	Toshiba TOSCANER-30000μCM ^e^	3D point clouds	Surface deviation (RMSE)	In reference to standard computed tomography imaging, data acquisition with a smartphone for 3D modeling is not as accurate as commercially available laser scanning.
Fourie et al. (2011) [47]	To estimate the reliability of three different 3D scanning systems namely laser surface scanning (Minolta Vivid 900), CBCT, 3D SPG (Di3D system) and to compare them to physical linear measurements.	Di3D ^a1^, Vivid 900 ^c^, KaVo 3D exam ^e^	7 cadaver heads	Direct anthropometry	15	21 linear distances (mean linear deviations)	Measurements recorded by the three 3D systems appeared to be both sufficiently accurate and reliable.
Gibelli et al. (2018) [48]	To compare the accuracy of SPG with a low-cost laser scanner.	Sense ^c^	50 participants (10 M, 40 F), 1 MH	Vectra M3 ^a1^	17; 3D point clouds	14 linear distances, 12 angles/volumes/surfaces (RMSE)	The low-cost laser scanner appeared sufficiently reliable for immovable objects but it is not suitable for 3D human face scanning.
Gibelli et al. (2018) [49]	To validate VECTRA H1 portable SPG device to verify its applicability to 3D facial analysis.	Vectra H1 ^a2^	50 participants (16 M, 34 F), 1 MH	Vectra M3 ^a1^	12; 3D point clouds	15 linear distances, 12 angles/volumes/surfaces (RMSE)	The portable Vectra H1 face scanning device proved reliable for assessing linear measurements, angles, and surface areas; conversely, the influence of involuntary facial movements on volumes and RMSE was higher compared to the stationary Vectra M3.
Kim et al. (2018) [50]	To evaluate the accuracy and reliability of a 3D small-format, handheld camera.	Vectra H1 ^a2^, Vectra M3 ^a1^	5 participants (NA)	Direct anthropometry	29	25 linear distances (mean linear deviations)	The 3D handheld camera showed high accuracy and reliability in comparison with traditional models.
Liu et al. (2019) [51]	To evaluate reliability of a portable low-cost scanner (Scanify) for imaging facial casts compared to a previously validated portable digital stereophotogrammetry device (Vectra H1).	Scanify ^a2^	2 IC (male)	Vectra H1 ^a2^	13	11 linear distances (mean linear deviations)	Scanify showed to be a low-cost solution for facial digitalization but needs future improvements.
Liu et al. (2021) [52]	To evaluate the reliability of Bellus3D Face Camera Pro compared to 3dMDface stereophotogrammetry for face scanning.	Face Camera Pro Bellus ^b3^ (connected to a tablet), 3dMD face ^a1^	1 MH	Direct anthropometry	20	8 linear distances, 5 angles (absolute mean deviations)	Both systems showed good accuracy and precision for clinical purposes.
Modabber et al. (2016) [53]	To evaluate the reliability of two devices for face digitalization.	Artec EVA ^d^	41 participants (16 M, 25 F)	FaceScan 3D ^a1^	2 lego brick	Surface deviation (RMSE)	Artec EVA showed greater accuracy compared to FaceScan3D.
Nightingale et al. (2020) [54]	To test the use of a low-cost scanner by inexperienced operators.	iPhone 8S ^b2^ (40, 60 or 80 photographs)	20 participants (11 M, 9 F)	Artec Spider ^d^	3D point clouds	Surface deviation (RMSE)	Smartphone photogrammetry showed to be useful for novice operators for its low cost and easy learning curve.
Piedra-Cascon et al. (2020) [55]	To evaluate the reliability of Bellus3D Face Camera Pro for face digitalization.	Face Camera Pro Bellus ^b3^ (connected to a tablet)	10 participants (2 M, 8 F)	Direct anthropometry	6	5 linear distances (precision and accuracy RMSE)	The new device showed to be clinically reliable for face scanning procedures.
Ross et al. (2018) [56]	To estimate the performance of smartphones for external ear digitalization.	iPhone 7 ^b2^ (30, 60 or 90 photographs), Intel RealSense Camera SR300 ^d^	16 participants (8 M, 8 F)	Artec Spider ^d^	6	Surface deviation (RMSE)	The three protocols with 30-60-90 photographs showed a good and similar accuracy. The Intel RealSense showed the worst performance and resolution.
Rudy et al. (2020) [57]	To compare the accuracy of portable stereophotogrammetry with iPhone X for facial digitalization.	iPhone X ^b1^	16 participants (NA)	Vectra H1 ^a2^	10; 3D point cloud	Landmark-to-landmark surface distances; surface deviation (RMSE)	The iPhoneX accuracy stood around 0.5 mm when compared to Vectra H1.
Wang et al. (2022) [58]	To compare the accuracy of two portable systems and a stationary scanner for facial digitalization.	iPad Pro 2020 ^b2^ (Bellus 3D Dental Pro app), EinScan Pro 2X Plus (EP+) ^c^, ARC-7 Face Scanning System ^d^	20 participants (5 M, 15 F)	Direct anthropometry	12	14 linear distances (absolute error)	All the systems showed to be quite reliable for face digitalization. However, the iPad Pro 2020 system was the least accurate.
Ye et al. (2016) [59]	To assess the accuracy, reliability and reproducibility of facial digitalization through stereophotogrammetry compared to a structured light scanner.	3D CaMega ^d^, 3dMDface^a1^	10 participants (5 M, 5 F)	Direct anthropometry	16	21 linear distances (mean linear deviations)	Both scanners showed to be quite accurate, reliable and reproducible to create 3D facial models.
Zhao et al. (2017) [60]	To evaluate the accuracy of different scanning systems for face digitalization.	3dMDface^a1^, FaceScan3D ^a1^	10 participants (NA)	Faro Edge LLP ^c^	3D point clouds	Surface deviation (RMSE)	All scanning systems showed to be sufficiently reliable for clinic purposes.
Zhao et al. (2021) [61]	To evaluate the accuracy of stereophotogrammetry and a CBCT system to obtain facial models with deformities and partitions.	3dMDface^a1^, NewTom 5G Inc ^e^	60 participants (28 M, 32 F)	Coordinate-measuring Machines (CMM)	17	19 linear distances (mean linear deviations)	3D stereophotogrammetry was more than CBCT in the acquisition of facial deformities.

**Table 5:** Comparison of stationary/portable stereophotogrammetry and smartphone scanning face systems.

Parameters	Stationary Stereophotogrammetry	Portable Stereophotogrammetry	Smartphones
Mean accuracy	0.087–0.860 mm	0.150–0.849 mm	0.460–1.400 mm
Costs	8.000–26.000$	1.500–10.000$	350–1.200$
Capture time *	1.5–600 ms	3.5–250 ms	2.5–350 ms
Approach	Different cameras placed at different angulations capture various 2D images simultaneously to create a 3D face model.	A single camera capture one image at a time. Require several acquisitions from different angles to reconstruct the 3D facial model.	A single camera capture one image at a time. Require several acquisitions from different angles to reconstruct the 3D facial model.
Advantages	Highly reliable system. Useful in children and uncooperative patients.	Reliable and portable system.	Comfortable, manageable, quite reliable, easy to use, inexpensive.
Limitations	Expensive, requires space and ambient light control.	Expensive, ambient light control, motion artifacts, greater acquisition time, require patient collaboration.	Motion artifacts, less accurate on uneven and deep surfaces, greater acquisition time, require patient collaboration.

* It refers to a single scan.

## Data Availability

Not applicable.
